# Emergency Response System for Pollution Accidents in Chemical Industrial Parks, China

**DOI:** 10.3390/ijerph120707868

**Published:** 2015-07-10

**Authors:** Weili Duan, Bin He

**Affiliations:** Key Laboratory of Watershed Geographic Sciences, Nanjing Institute of Geography and Limnology, Chinese Academy of Sciences, Nanjing 210008, China

**Keywords:** emergency response system, chemical industrial parks, pollution accidents, domino effects, emergency rescue

## Abstract

In addition to property damage and loss of lives, environment pollution, such as water pollution and air pollution caused by accidents in chemical industrial parks (CIPs) is a significant issue in China. An emergency response system (ERS) was therefore planned to properly and proactively cope with safety incidents including fire and explosions occurring in the CIPs in this study. Using a scenario analysis, the stages of emergency response were divided into three levels, after introducing the domino effect, and fundamental requirements of ERS design were confirmed. The framework of ERS was composed mainly of a monitoring system, an emergency command center, an action system, and a supporting system. On this basis, six main emergency rescue steps containing alarm receipt, emergency evaluation, launched corresponding emergency plans, emergency rescue actions, emergency recovery, and result evaluation and feedback were determined. Finally, an example from the XiaoHu Chemical Industrial Park (XHCIP) was presented to check on the integrality, reliability, and maneuverability of the ERS, and the result of the first emergency drill with this ERS indicated that the developed ERS can reduce delays, improve usage efficiency of resources, and raise emergency rescue efficiency.

## 1. Introduction

During recent years, accompanied with the high-speed development of petroleum and chemistry industries, chemical industrial parks (CIPs) are today perceived as an integral part of development strategies of many countries worldwide. According to UNEP (1997) [1], among developed countries, there are approximately 8800 industrial parks in the US, 1200 in Canada, 200 in the UK, and 300 in Germany. The Houston industrial park, in America, Nordrhein-Westfalen industrial park, in Germany, Haute-Normandie industrial park, in France, and Antwerp industrial park, in Belgium, *etc.*, are now well-known chemical industrial parks and have created a better economy for these countries. As of today, according to incomplete statistics from the Ministry of Land and Resources, there are 6866 industrial parks in China spread across the whole country, of which 113 were government-approved national, provincial, and municipal parks [2]. CIPs offer a range of advantages over large petroleum and chemistry enterprises, as minimal infrastructure and utilities cost, complimentary industries, and services can further initiate multiplier effects on the surrounding region. Thus, properly planned CIPs can bring the benefit of scale economics and accelerate regional development. Due to such advantages, industrial parks have played an important role in the national development strategies of many countries and have been irreplaceable where economic development is concerned.

**Figure 1:**
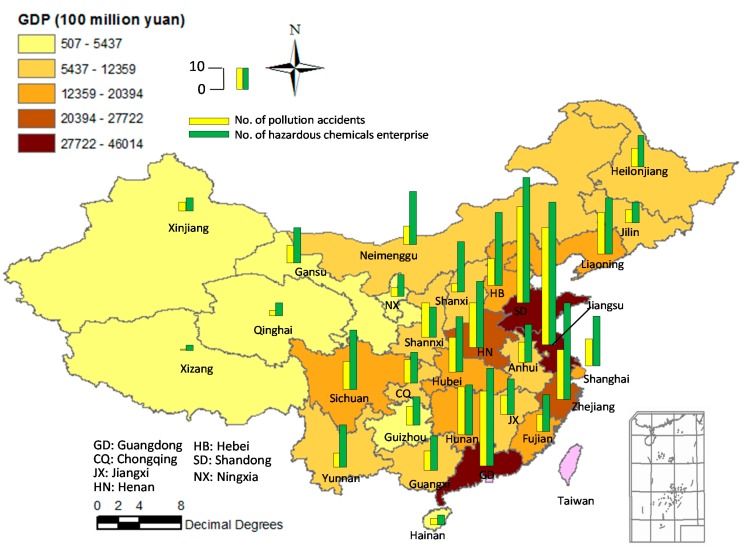
Sketch map of the relationships among the number of pollution accidents (700 pollution accidents happened from 1952 to 2010), gross domestic product (GDP, 2010), and the number of hazardous chemicals enterprises in China (Accident data from [8])

**Figure 2:**
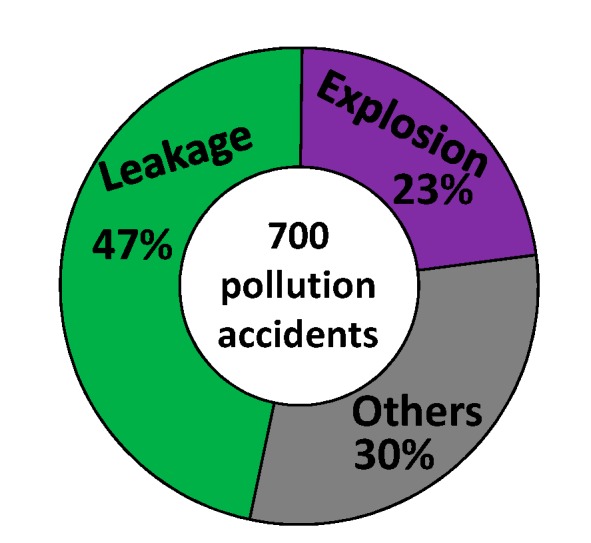
Causes of 700 pollution accidents that happened from 1952 to 2010 (Data from [8]).

However, CIPs are high-risk areas where many chemical plants are gathered. Due to the domino effect, once an accident occurs in CIPs, it may lead to grave accidents and casualties [3,4]. Meanwhile, environment pollution such as water pollution and air pollution caused by the domino accidents in CIPs is a significant issue for watersheds in China [5,6,7]. Figure 1 shows the relationships between the number of pollution accidents (1952–2010) [8], gross domestic product (GDP, 2010), and number of hazardous chemicals enterprise in China, suggesting that the pollution accident rate in developed southeast coastal areas, e.g., Guangdong, Jiangsu, and Shandong, was far higher than that in the northwest areas, e.g., Xinjiang, Xizang, and Qinghai. Figure 2 shows the causes of about 700 pollution accidents that happened from 1952 to 2010, revealing that hazardous materials leakage and explosions were the main causes of pollution accidents, up to 47% and 23%, respectively. In recent years, the most serious water pollution incident was the Jilin Petrochemical benzene plant explosion, which occurred on 13 November, 2005, and the explosion severely polluted one of China’s biggest rivers, the Songhua River, with an estimated 100 tons of pollutants, containing benzene and nitrobenzene, entering into the river, which caused that water supplies to be cut and inaccessible for millions of people in cities along the river for various periods.

Therefore, effective emergency response is crucial for containing its impact to the smallest possible area around the accident site [9]. Countering this problem, several researchers have studied emergency response systems and most emergency systems have their own objectives, features, characteristics, and structures. Hill [10] established an Emergency Management Combined Response System, which can be used to diagnose inter-agency co-ordination problems. Tseng *et al.* [9] developed an adequate emergency response plan (ERP), with safety and industrial hygiene resources, to deal with the effects resulting from a chlorine gas leak, which can effectively lessen or avoid injury to plant personnel and citizens in the neighboring community. Using a Petri net, Zhong *et al.* [11] studied the performance of Urban Emergency Response Systems (UERS) and established its PN model for performance analysis. Using a critical incident management systems (CIMS) efficiency model, Kim *et al.* [12] developed an instrument to measure the critical factors that contribute to the efficiency of decision support in CIMS, which allows communities to assess both strengths and weaknesses of existing systems. In China, safety management for the chemical industry has made some achievements. However, judging from the accidents in recent years (e.g., the “12.23” Kaixian blowout accident [13], Jilin Petrochemical benzene plant explosion [14]), much of the existing ERS in China is not systematic and complete [15] and, therefore, this keeps levels of environmental management and safety management low [16]. Specifically, the disadvantages of ERS in China mainly consist of the following aspects: (a) Timeliness problems: the emergency rescue has not been carried out in a timely manner because ERS cannot get the effective information in time; (b) The decision-making blunders: due to lack of a perfect decision support system, the accident commander is likely to make wrong decisions; (c) Active disorders: sometimes, the organizational structure of ERS is not perfect and rescue teams are not in place. To address these problems, the present study is trying to establish a sound, comprehensive ERS to prevent the future accidents and minimize losses in CIPs in China.

The paper is organized as follows: using a scenario analysis, Section 2 provides an introduction to the domino accidents in CIPs. Section 3 builds a common framework model of ERS, which is composed, mainly, of a monitoring system, an emergency command center, an action system, and a supporting system. On this basis, Section 4 constructs the basic and practical procedures of the emergency rescue. In Section 5, we introduce and illustrate the application of the ERS in CIPs. Section 6 concludes this paper with the discussion of limitations and future research.

## 2. Accident Scenario Description and Fundamental Requirements of ERS Design

### 2.1. Scenario: Domino Accidents

**Figure 3:**
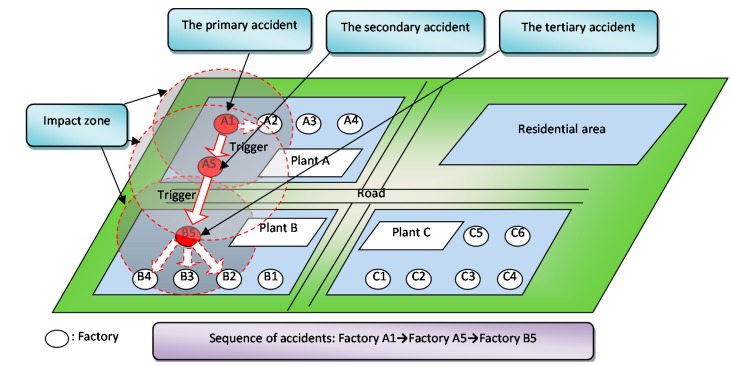
Scenario of domino chemical accidents.

Generally, “domino accident” means that one disaster leads to another, and often another, which may be initiated by one or more of these causative events [17,18,19,20]: (a) Fire: pool fire, flash fire, fireball, and jet fire; (b) Explosion: confined vapor cloud explosions (CVCE), boiling liquid expanding vapor explosion (BLEVE), vented explosion, vapor cloud explosion, and dust explosion; (c) Toxic release: instantaneous or continuous release of toxic light-as-air-gases; lighter-than-air gases, and heavier-than-air gases; release of toxic liquids. Moreover, because of the dangerous character of chemicals, fires, explosions, and toxic releases are occurring at the same time in CIPs. Therefore, we have developed a scenario involving a domino chemical accident. In this scenario we assume that, as illustrated in Figure 3, each accident creates an impact zone (the impact zones are illustrated as circular only for the sake of simplicity. Environmental effects such as wind or rain may change the shape and development pattern of an impact zone), within which the normal society’s activities are affected negatively [21].

### 2.2. The Level of Emergency Response

**Figure 4:**
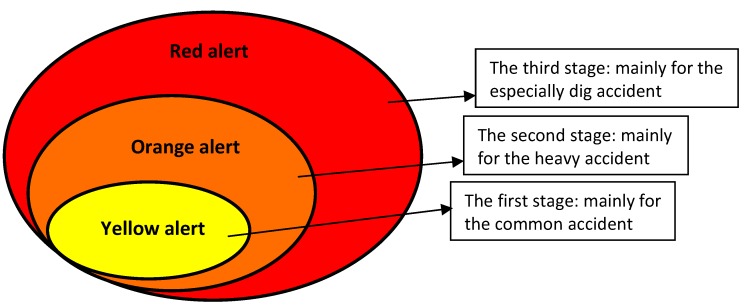
The relationship of the three stages of emergency responses.

According to the scene situation, response services prescribe the actions to take, policies to execute, and rules to follow. Generally, the emergency response services may include firefighting, rescue, decontamination, medical care, evacuation, securing the scene and the perimeter, and traffic control and law enforcement (to prevent lawlessness). The accident severity is the most important criterion to be considered for the emergency response and, thereby, three stages of the emergency response were established (Table 1 and Figure 4).

**Table 1:** The stage of emergency response.

No.	The Stage of Emergency Response	Signal	Emergency Response Actions
1	The first stage (plant level)	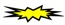 Yellow alert	The plant and its related department can control the accident by itself, even without further support from outside.
2	The second stage (CIP level)	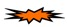 Orange alert	Emergency staff of CIPs can be called on to control the accident.
3	The third stage (region level)	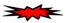 Red alert	As long as the accident needs other support from outside, the regional emergency response would be launched.

In CIPs, three kinds of accidents are possible. However, from the domino effect, and cases in the past, how to understand and deal with the heavy accident (or the especially large accident) is more sophisticated and important. In both situations, emergency response services may include restricted areas, firefighting, victim rescue, first aid, decontamination, security, resident evacuation, traffic, *etc.*

### 2.3. Fundamental Requirements of ERS Design

From the above analysis, the process of emergency management will become more and more complicated because of the domino effect. Time is of the essence in responding to chemical accidents [22], which requires that all emergency response services must be accessible. We summarized the fundamental requirements of ERS design (Table 2) according to the requirements of an effective emergency response architecture [23,24,25].

**Table 2:** Fundamental requirements of system design.

No.	Requirements	Description
1	Effective information collect, analysis and forecast (including monitoring system)	Emergency response data collection, compilation, analysis, and storage.
2	Directory of first response resource	Database of personnel, equipment, and tools with their availability, amount, and properties.
3	Knowledgebase of task related information	Police, legal regulation, code, reference, and maps.
4	Communication support	Multiple communication channel, mobile, robust, and secure communication
5	Decision making support(including Defense Security Service)	Autonomic decision making, decision role delegation, and expert system
6	Response tracking support	Updating and tracking of personnel location, resource assumption, and task progress
7	Multimedia support (including Geographic Information System, Photographic System of Wireless Networks)	Visualization tools for representing, decision making, and communication
8	Security support	Secured information flow and access control
9	Fault tolerance and redundancy support	Data backup, distributed data storage, load balance, and mirrored hot servers

## 3. Framework for the ERS

**Figure 5:**
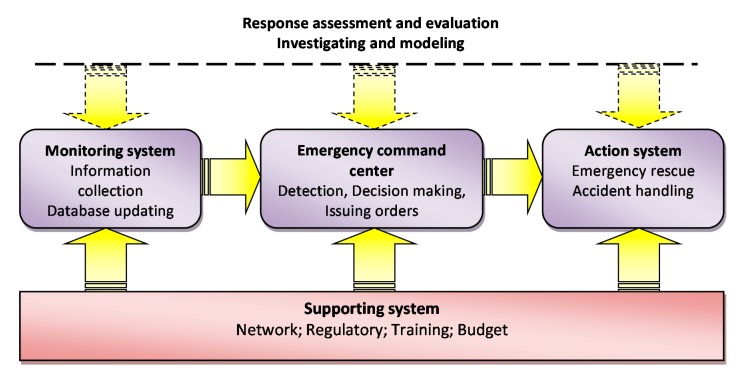
Framework of the emergency response system for CIPs.

Analysis of the accident scenario and fundamental requirements indicates that the emergency rescue should have an orderly course for every step, including identifying and confirming, fast reaction, information, accident management, accident area control, *etc.* The factors that affect the emergency response to domino accidents in CIPs are various and their mutual relationships are complicated. Thus, a proper emergency information database should be established to monitor the dynamic changes of CIPs in real time using advanced information technology, control technology, network information technology, *etc.* In this study, the ERS in CIPs is composed of four parts: (1) a monitoring system, (2) an emergency command center, (3) an action system, and (4) a supporting system. Figure 5 shows the relationships between the four components and their functional characteristics. The monitoring system captures chemical accident emergencies and reports to the emergency command center, which assesses the emergency, raises alerts, and makes decisions to respond to the emergency. The action system receives commands from the emergency command center and carries out the emergency response. The supporting system serves as a compulsory force to ensure all the activities of the ERS are undertaken properly. Lessons learned during, and after, an accident will be incorporated into the body of knowledge of improving the ERS and reflected in future emergency detection and response. Overall, the ERS is an integrated system that can be installed, run and integrated through wireless and other networks on wireless laptops, accident command vehicle-mounted desktops, and the emergency command center.

### 3.1. Monitoring System

**Figure 6:**
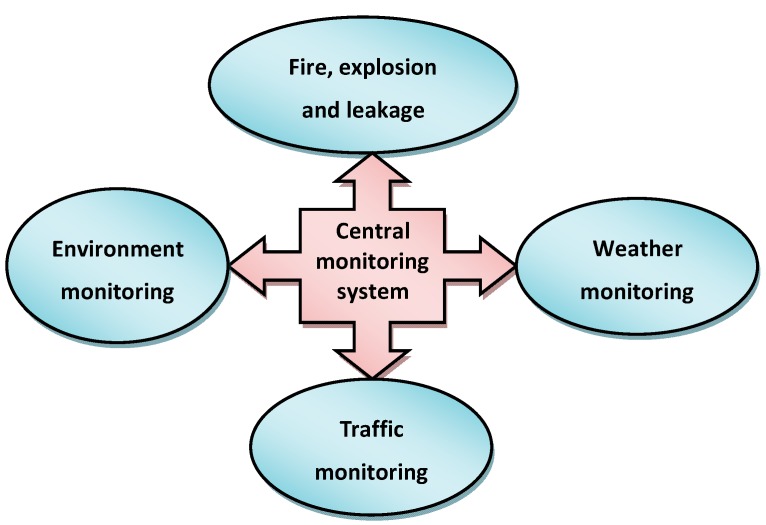
Elements of the monitoring system.

The monitoring system is an important component of the ERS, the purpose of which is to collect all types of information about the multiple hazards of CIPs. That is, the monitoring system can integrate all of the safety apparatus relative to life, property, and the public to keep the staff from danger. Especially when an accident occurs, it can provide real-time information for the emergency rescue as early as possible, including the accident location and situation, environmental, weather, and climate data [26]. Here the monitoring system is mainly composed of four subsystems (Figure 6).

(1) Fire, explosion and leakage monitoring subsystem

This subsystem is set up in each plant in CIPs, and the data and information of the chemical leakage, fire, and explosion are reported to the central monitoring system. Concretely, once an accident occurs, the monitoring staff will receive the information and the accident and situation will be handled simultaneously.

The functions of the fire, explosion, and leakage monitoring subsystem include the following factors:

(a) The accident location and situation could be viewed by choosing the damaged area within its plant.

(b) The location and conditions of the area and plant can be selected to relieve the victims in a disaster area and provide emergency assistance.

(c) Users could enter the screen of monitoring with permission.

(2) Environment monitoring subsystem

Hazardous chemical accidents often lead to negative consequences for the environment, which is an issue of serious international concern. Monitoring of environment pollution is an important linkage in accident handling, a pressing task. Its functions should include two main items:

(a) Monitoring of environment, water, and compressed air systems to ensure they meet the environmental standards.

(b) Geographical and topographical of CIPs.

(3) Weather monitoring subsystem

Weather variation is an important factor affecting the emergency rescue [27]. Rescue efforts will be severely hampered if extreme weather (e.g., typhoon, rain storm, *etc.*) happens. Usually, the weather monitoring subsystem is founded upon the local weather bureau, and its functions contain the following aspects:

(a) Rainfalls or torrents are seriously considered, and the data are renewed from the computer on a daily basis.

(b) The main computer is operated in coordination with stand-by and redundant systems.

(c) The data of rainfalls are displayed on the screen in the weather monitoring subsystem.

(d) Wind speed, wind direction, stability conditions, cloud cover, temperature and relative humidity.

(4) Traffic monitoring subsystem

Through the traffic monitoring subsystem, the monitoring center to keeps abreast of the situation of road traffic. By analyzing the traffic volume, speed, and occupancy using the method of abnormal automatic traffic detection, we can distinguish whether the road is jammed or not, and the causes, and whether abnormal traffic has occurred or not, and the site and severity. According to this analysis, the emergency rescue team can find which route is the best to evacuate people.

### 3.2. Emergency Command Center

A critical part of the ERS involves preparing to operate an emergency command center. Good response and recovery management requires a robust approach to information management. Command centers, supported by sound information management systems, hold the key to successfully managing potential problems associated with any emergency rescue [28]. Simply put, it intends to utilize advanced information technology to provide critical information to a central command team so that appropriate decisions can be made to deal with the chemical accident emergency rescue. The system will monitor the entire process of the emergency and has such functionalities as information translation, crisis determination, decision support, command, deployment, real time communication, and onsite support. The purpose is to make the most appropriate response to the emergency in the shortest time so that available resources can be effectively and efficiently allocated and exploited. The systems in an emergency command center mainly include three application platforms and six subsystems. The three application platforms are the information platform, professional service platform, and decision making platform. The six subsystems include the database subsystem, analysis and prediction subsystem, virtual reality subsystem, decision support subsystem, search engine subsystem, and accident command subsystem. Since the emergency command center is the central component of the emergency response system, the three platforms and six subsystems are described in more details in the following three paragraphs.

The information platform consists of the development of computer software and hardware, data preprocessing system, and database. In this platform, the initial data from the monitoring (e.g., accident location and its situation, environment data, weather data, traffic data) are processed and then loaded into the database.

Data storage technologies include both centralized and distributed database management models. The professional service platform is the core of the command center. Employing simulation techniques, this platform builds models (e.g., accident consequence modeling) to analyze the spreading processes of the chemical accident. This exercise is largely based on the accident model database, the methodology database, and the accident causation theory. The decision-making platform is the presentation level of the command center. All the activities involved in the chemical accident emergency response, which includes analyzing, investigating, predicting, decision-making, executing, and getting feedbacks, are carried out at this level.

**Figure 7:**
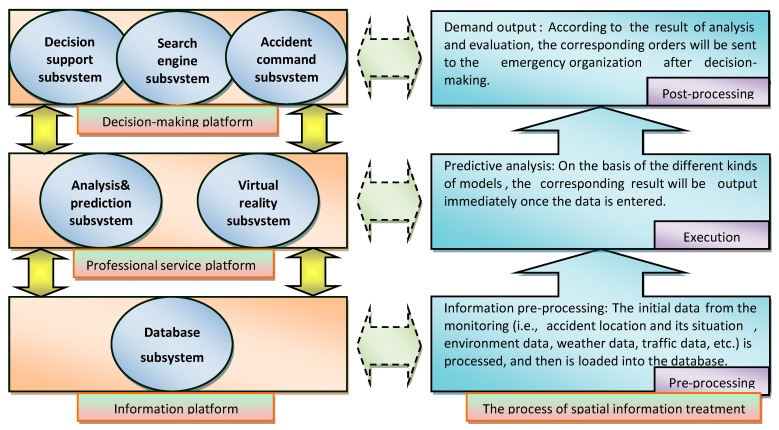
Elements of the emergency command center and the process of spatial information treatment.

The six subsystems provide technical support to three application platforms. As shown in Figure 7: (1) the database subsystem supplies data to the information platform, (2) the analysis and prediction subsystem and the virtual reality subsystem serve the professional service platform, and (3) the decision support subsystem, the search engine subsystem and accident command subsystem offer services to the decision-making platform. The three platforms and the six subsystems need to share information or exchange data on a regular basis. They are integrated together to support the chemical accident emergency response and their performances are related with each other.

### 3.3. Action System

**Figure 8:**
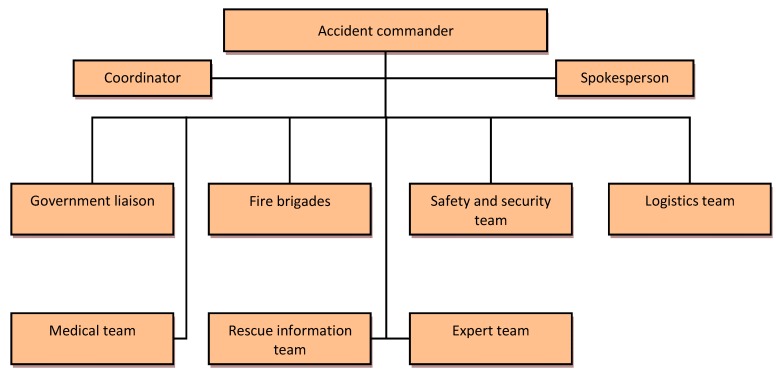
The organizational structure of the action system.

As the name suggests, the action system consists of all kinds of rescue behaviors (e.g., victim rescue, personal evacuation, firefighting, chemical materials disposal, medical service, materials supply), and the implementation is the main responsibility of rescue teams. Thus, the organizational structure of ERS is the heart of the system. Given the complexity and variety of rescue behaviors, a sound organizational structure must include all kinds of emergency rescue teams. Figure 8 depicts the organizational structure of the action system.

Simultaneously, a scientific and complete organizational structure must also have a clearly defined responsibility system. That is, each rescue organization must have clear responsibilities, clear objectives, and tasks to ensure that the emergency rescue would achieve good results. Thus, based on the different roles at different stages in the emergency rescue [29,30], we have developed a reasonable task table, as is shown in Table 3.

### 3.4. Supporting System

The supporting system ensures the ERS is running properly and stably. Its main task is to realize the data exchange among the systems and share information. Thus, the supporting system requires the establishment of emergency response LANs and central databases at the plant, CIPs, and region levels. This network system introduces local network technology in management, archives information integration, and resource sharing of various control systems of the whole emergency response system. At the same time, according to the difference of visitors’ identities, users can access the corresponding functions. In addition, with the development of technology, the corresponding standardized format of regulatory reports will be improved.

**Table 3:** Job duty of each rescue organization.

NO.	Emergency Rescue Organization	Work Assignment
1	Accident commander	Activating elements of the emergency response system Executing and planning the emergency response actions Initiating the evacuation order to the staff Assigning manpower resources Approving requests for additional resources and requests for the release of resources
2	Coordinator	Coordinating the rescue team and offering the response measures Monitoring the incident operations to identify what might be potential inter-organizational problems Bridging between the incident commander and rescue team for assisting to dispatch each task Coordinating the task on
3	Spokesperson	Issuing and explaining the incident information Explaining the status of the emergency response process Setting up and participating in a press conference
4	Government liaison	Contacting and reporting information to related governmental agencies Contacting the department of emergency command center to request safety and health equipment for other departments to use to control the upset situation
5	Fire brigades	Rescuing people in danger Controlling fires
6	Safety and security team	Guiding and evacuating the staff and vehicles Safely guiding the support-personnel into the plant Evacuating visitor and onlookers to a safe location Closing off the scene of the accident
7	Medical team	Providing first aid and transporting the injured to a hospital Arranging for medical supplies Alerting the nearby hospital of potential patients
8	Rescue information team	Providing and checking out the safety and health equipment Recording rescue information Assisting the incident analysis Environmental and weather analysis
9	Expert team	Participating in the development of an incident action plan and review the general control objectives, including alternative strategies as requested Collecting and transmitting records and logs to the documentation unit at the end of each operational period
10	Logistics team	Providing logistical support of all kinds to field forces Coordinating and processing requests for additional emergency resource

## 4. Workflow of the ERS

**Figure 9:**
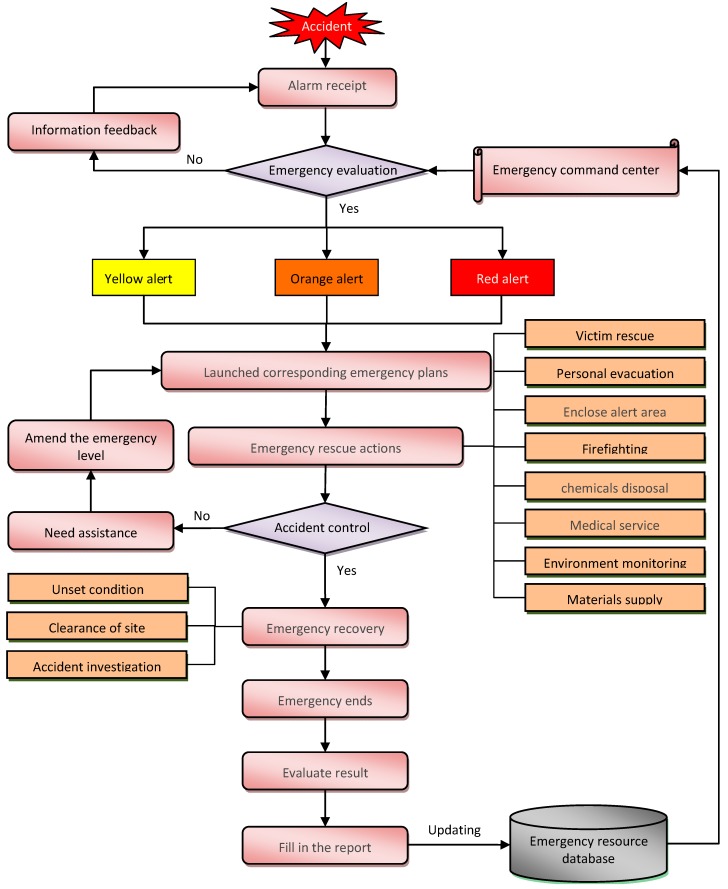
Emergency response steps for chemical accidents of CIPs.

Under the framework of the ERS, the implementation of the emergency response is a complicated project [30]. Therefore, it is very important to analyze data and determine corrective actions following the credible scenario of the chemical accident of CIPs. Moreover, based on the domino accident scenario (see Section 2), we can conclude that the basic emergency procedures of three different levels are the same, excepting different-sized responses. Six main steps are used to carry out the emergency rescue process (Figure 9).

Step 1: Alarm receipt

Once an accident has occurred, workers in the monitoring center find the accident in the plant covered by the monitoring system, immediately. At the same time, the accident victims or witnesses can raise the alarm on the emergency telephone in the accident region or by cell phone.

Step 2: Emergency evaluation

After receiving an accident alarm, the emergency command center immediately records the accident information, including the time, place, accident type, and accident description, then makes a preliminary analysis and confirmation, and the emergency rescue pre-project will be generated automatically. Simultaneously, the accident information will be issued through the monitoring system and supporting system.

Step 3: Launch corresponding emergency plans

After confirming the accident type and the accident region, the accident commander will launch the corresponding emergency plan through the emergency command center.

Step 4: Emergency rescue actions

Then, all emergency rescue orders will be issued through the emergency command center and the emergency rescue project will be carried out. According to the different rescue requirements and functions of teams, the accident and rescue requirement information will be distributed to all the relevant teams which are involved in the rescue. Sometimes, although the emergency rescue team shall actively adopt salvaging measures so as to prevent elevation of its seriousness in the early stage of emergencies, the impact of the domino accident is still unfolding. Thus, when the accident cannot be controlled, it is necessary to increase the rescue forces.

Step 5: Emergency recovery

After rescuing victims and controlling the accident, the accident commander will declare a state of emergency recovery. Its major tasks are: first, to unset the dangerous condition; second, to clear the accident site; third, to investigate the accident; finally, the incident commander, by various approaches, needs to placate the neighboring citizens and reassure the victims.

Step 6: Evaluate result and feedback

The emergency command center issues an order that the emergency response has ended when the rescue action is finished. The commander needs to thank all of the members who executed the ERS, and then request the spokesperson to hold a press conference to explain the whole process of handling the accident. Meanwhile, the emergency command center will record a detailed accident rescue treatment and evaluate the treatment result. On this basis, the emergency command center will update the emergency resource database and to send feedback for the emergency command center to improve upon. All of the above-mentioned steps must be rehearsed monthly in order to lessen the degree of hazard if an accident occurs.

## 5. Application

**Figure 10:**
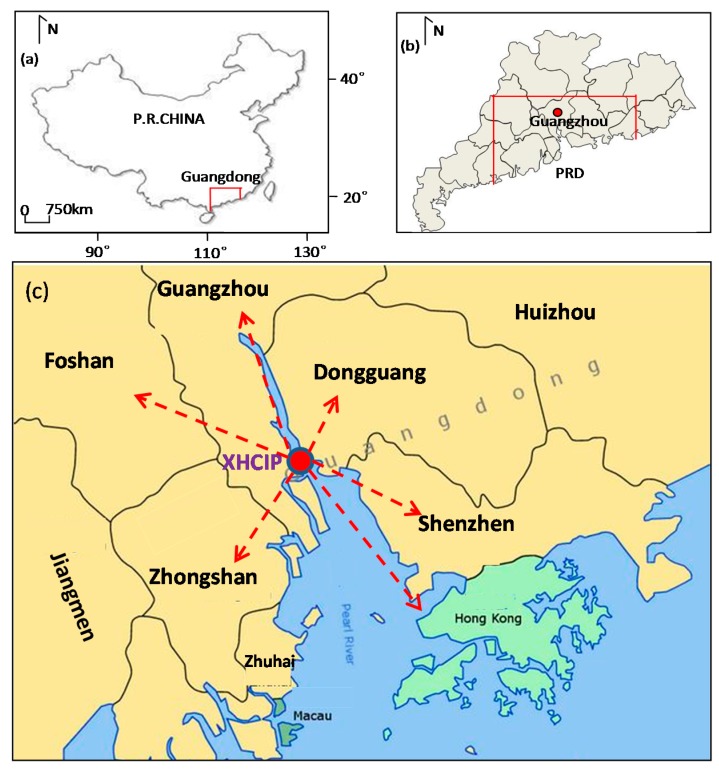
Schematic showing the geographical locality of (**a**) the Guangdong Province in China; (**b**) Guangzhou city in the Guangdong Province and Pearl River Delta (PRD) region; and (**c**) the XiaoHu Chemical Industrial Park (XHCIP) in Guangzhou Province.

The XiaoHu Chemical Industrial Park (XHCIP) is located in the economic, traffic and transportation, well-developed Pearl River Delta (PRD) region of Guangdong Province—Guangzhou’s Naamsa District, 36 km from the urban area of Guangzhou, Hongkong, 67 km, a total area of 97,000 square meters (Figure 10), and a total of 38 domestic and foreign chemical enterprises (Data was collected in 2011) have settled, so far. Obviously, once there is a chemical domino accident, it is quite possible that the chemical contamination causes great negative impact in the environment and society of its surrounding areas. Thus, the XHCIP is becoming a key area for the country’s chemical industry production safety supervision.

**Figure 11:**
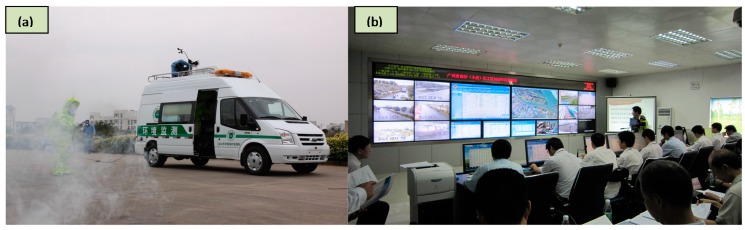
Photographs showing some parts of ERS: (**a**) the environmental monitoring car is used to detect the hazardous gas with toxic gas sensors; (**b**) accident commander is directing rescue operations in the emergency command center of XHCIP.

In order to improve the emergency management, and to reinforce emergency system construction, the ERS has basically taken shape in XHCIP at present. The system mainly consists of the following parts: First, a real-time monitoring system of major hazard sources have been gradually established, which includes many humidity, temperature, pressure, and concentration detectors, camera monitors, and monitoring vehicles (Figure 11a); Second, an emergency command center has been constructed (Figure 11b); Third, a perfect organizational structure has been set up, which is composed of relevant departments of the municipal government (e.g., safety management, public security, environmental protection, public health departments, *etc.*). Moreover, with the development of new technology in safety management, the ERS of XHCIP is also improving.

In order to check on the integrality, reliability, and maneuverability of the ERS, the first emergency drill was carried out on 25 June 2010. The accident scenario was that a large fire broke out suddenly in a plant in XHCIP due to electrostatic charges in the course of acetone unloading, and the specific process of emergency rescue is similar to that of Section 4. Comparing with the former rehearsals (e.g., 12 December 2008), this emergency drill has the following advantages: (1) Reduce the emergency response time of accidents. Although these rehearsals are on a different scale, we found the availability of the ERS obviously reduced the emergency response time of accidents. For example, the time of alarm receipt was shortened. (2) Raise the efficiency and accuracy of emergency rescue actions, including stopping the growth and spread of fire. The use of the ERS proved to be an effective tool for emergency management, e.g., real-time data collection, estimation of impacted area, determination of the candidate evacuation routes, and for helping the decision makers to visualize the modeling results. All of this improves the usage efficiency of resources and raises the efficiency and accuracy of emergency rescue actions.

## 6. Conclusions and Future Work

In this paper, according to the accident scenario, we have established an ERS to properly and proactively cope with incidents occurring in CIPs, which is composed mainly of a monitoring system, an emergency command center, an action system, and a supporting system. It involves information technology, network technology, safety management, intellectual technology, hazardous waste management, and usage. Moreover, all technologies and methods are unified as a whole in different systems, which makes up the particular structures and functions. If we take the ERS as a body, then its monitoring system is equivalent to the human sensory system, the main task is to collect and update information; its emergency command center serves as the nervous system, to translate perception information into action instructions; its action system, like the human movement system, to execute instructions; its support system corresponds to the body’s other systems to provide support for the above processes. At the same time, according the framework of the ERS, steps of the emergency rescue have been determined, which is mainly composed of six steps: alarm receipt → emergency evaluation → launched corresponding emergency plans → emergency rescue actions → emergency recovery → evaluate the result and feedback. Finally, an example from the XHCIP is presented to check on the integrality, reliability, and maneuverability of the ERS, and the result of the first emergency drill shows that the ERS can reduce delays, improve usage efficiency of resources, and raise emergency rescue efficiency. Of course, in actual operation, the emergency response will be more complex. Thus, to complete a comprehensive, useful and realistic system, all workers are welcome to provide their views and suggestions to the overall ERS to avoid the unfit and unreal situations. In addition, table-top or functional exercises should be implemented monthly, semi-annual or yearly. Only through the reviews of exercises and real accidents, a fail-safe and real-life system could be established to lessen the hazards in CIPs.

The current research does suffer from several limitations. First, we mainly discuss the accident scenario involved in the chemical accident (*i.e.*, fire, explosion, and toxic release) but not the natural catastrophes (e.g., tornadoes, earthquakes, and floods) [31,32]. Yet, most CIPs are located in the southeast coast of China, which often faces natural catastrophes. Second, in actual operation, the emergency rescue will be more complex, and therefore it is possible that the ERS will not work in a normal condition in the event a subsystem is destroyed (e.g., the sensor of monitoring system may be destroyed in fire and explosion).

The management of ERS is an important, but difficult, research topic. The improvement of ERS requires collective and continuous research efforts to enhance the body of knowledge and to improve the “best practice”. To further our research, we are interested in the following future research directions: (1) an investigation into the natural catastrophes and the development of integrated systems in CIPs, (2) the development stages of emergency management technology for CIPs, (3) the proposal of a performance metric for ERS evaluation purposes, and (4) a training and exercising system to provide realistic command training and exercising for common emergency scenarios that is highly cost-effective.
